# Next-generation sequencing of dsRNA is greatly improved by treatment with the inexpensive denaturing reagent DMSO

**DOI:** 10.1099/mgen.0.000315

**Published:** 2019-11-18

**Authors:** Alexander H. Wilcox, Eric Delwart, Samuel L. Díaz-Muñoz

**Affiliations:** ^1^​ Department of Microbiology and Molecular Genetics, University of California, Davis, CA, USA; ^2^​ Vitalant Research Institute, San Francisco, CA, USA; ^3^​ Department of Laboratory Medicine, University of California San Francisco, San Francisco, CA, USA; ^4^​ Genome Center, University of California, Davis, CA, USA

**Keywords:** dsRNA, viral sequencing, innate immunity, metagenomics, dsRNAseq, sociovirology

## Abstract

dsRNA is the genetic material of important viruses and a key component of RNA interference-based immunity in eukaryotes. Previous studies have noted difficulties in determining the sequence of dsRNA molecules that have affected studies of immune function and estimates of viral diversity in nature. DMSO has been used to denature dsRNA prior to the reverse-transcription stage to improve reverse transcriptase PCR and Sanger sequencing. We systematically tested the utility of DMSO to improve the sequencing yield of a dsRNA virus (Φ6) in a short-read next-generation sequencing platform. DMSO treatment improved sequencing read recovery by over two orders of magnitude, even when RNA and cDNA concentrations were below the limit of detection. We also tested the effects of DMSO on a mock eukaryotic viral community and found that dsRNA virus reads increased with DMSO treatment. Furthermore, we provide evidence that DMSO treatment does not adversely affect recovery of reads from a ssRNA viral genome (influenza A/California/07/2009). We suggest that up to 50 % DMSO treatment be used prior to cDNA synthesis when samples of interest are composed of or may contain dsRNA.

## Data Summary

Sequence data was deposited in the National Center for Biotechnology Information Short Read Archive (accession numbers: PRJNA527100, PRJNA527101, PRJNA527098). Data and code for analysis is available at GitHub (https://github.com/awilcox83/dsRNA-sequencing/) (https://dx.doi.org/10.5281/zenodo.3530803). The protocol for dsRNA sequencing has been posted on protocols.io (http://dx.doi.org/10.17504/protocols.io.ugnetve).

**Impact Statement**

dsRNA is the genetic material of important viruses and a key component of RNA interference-based immunity in eukaryotes. However, it is difficult to determine the genetic sequence of this critical nucleic acid using new high-throughput sequencing techniques. We developed a simple protocol using a cheap reagent that dramatically improves dsRNA sequencing coverage without affecting accuracy and sequencing of other RNA molecules. This method will impact three areas important for research into human, animal, agricultural and ecological health, by enabling sequencing of: (i) dsRNA viruses known to infect humans, animals, plants, fungi and bacteria; (ii) key dsRNA molecules that mediate human, animal and plant defences against pathogens through the immune system; and (iii) undiscovered viruses in our microbiomes and other natural and engineered environments.

## Introduction

RNA is a ubiquitous biological molecule involved in transcription and translation, which also serves as the genetic material of a large number of important viruses. The double-stranded form of RNA (dsRNA) is believed to be less abundant in nature, but is a crucial component of a number of biological systems. It has a central role in the RNA interference system [1], which modulates innate immunity in plants and animals, and serves as a replicative intermediate of (+) ssRNA viruses, while also being present in dsDNA and (−) ssRNA infections [2]. Moreover, it also serves as the genetic material of a number of virus lineages (including the families *Reoviridae*, *Cystoviridae* and *Picobirnaviridae*) that infect humans, animals, plants, fungi and bacteria, which play important medical, ecological and scientific roles. A number of dsRNA viruses are of clinical and agricultural significance, such as Bluetongue virus, which causes high morbidity and mortality in ruminants [3], and rotavirus, which causes acute gastroenteritis in humans [4]. There are indications that the overall diversity of RNA viruses may be underestimated [5, 6] and difficulties sequencing dsRNA in particular have been noted in the literature [7, 8]. As a consequence, dsRNA virus lineages may be under-represented and dsRNAs involved in immunity may be underestimated.

## Theory and implementation

The extent of microbial diversity has been revealed by powerful whole-genome sequencing tools that are quickly becoming standard tools in biology. However, next-generation sequencing has known biases according to the nucleic acid composition [9]. A major limitation is that most sequencing platforms cannot sequence RNA directly, requiring that it is first reverse-transcribed into its cDNA. cDNA synthesis is typically achieved by hybridizing oligonucleotide DNA primers to the RNA and using a reverse transcriptase to synthesize the remainder of the cDNA strand. This step poses a particular problem to dsRNA, because the presence of a complementary strand blocks the ability of these primers to bind. The blocking has a direct effect on the amount of dsRNA converted to cDNA, resulting in many fewer sequencing reads being generated relative to the true amount of RNA present. Additionally, many dsRNA viruses have small genomes that limit the amount of RNA that can be extracted, further complicating the determination of dsRNA sequences.

As early as 1968, DMSO had been shown to have a denaturing effect on nucleic acids [10]. DMSO has been successfully used to improve the performance of reverse transcriptase PCR [11] and Sanger sequencing [12]. However, to our knowledge DMSO has not been used for next-generation sequencing approaches and many dsRNA sequencing studies omit DMSO treatment [7, 13–15]. Moreover, there is no standard protocol for DMSO treatment of samples and previous methods vary greatly in their conditions, particularly DMSO concentration, which has ranged from 15 % [12] to 90 % [16].

This work investigated four questions regarding the effect of DMSO treatment on next-generation sequencing. (i) Does DMSO treatment improve recovery of dsRNA reads and at what concentration? (ii) Does DMSO affect read coverage and accuracy of a viral genome? (iii) Is the effect of DMSO independent of RNA concentration? (iv) Does DMSO treatment negatively affect the recovery of ssRNA genomes? Our results suggest that treatment with a high concentration of DMSO greatly increases the number of reads generated when sequencing dsRNA with no effect on read accuracy, without adversely affecting sequencing of ssRNA virus genomes on the Illumina short-read sequencing platform.

We carried out the methods in this paper on two different viruses: *Pseudomonas* phage Φ6, which has a dsRNA genome made up of three segments, and human influenza virus A H1N1, which contains eight ssRNA segments. We also used a mock eukaryotic viral community, manufactured by the National Institute for Biological Standards and Control (NIBSC, UK), as a reference material for multiplex viral detection (NIBSC reagent 11/242–001). This reagent was expected to contain 25 human pathogenic viruses and has been used to investigate viral detection methods on mixed and metagenomic samples [17].

### Sample preparation

Φ6 lysate was prepared by plating the phage and its *Pseudomonas syringae* host using double agar overlay. Phages were harvested by selecting a plate with semi-confluent lysis, transferring the soft agar layer to 3 ml LB (Lennox) media, and centrifuging to remove host cells and agar. An influenza virus lysate was generated from an egg-passaged stock of influenza A/California/07/2009 (H1NI) (generously provided by Ted M. Ross, University of Georgia, GA, USA), which was expanded by passage at a low m.o.i. in Madin–Darby canine kidney (MDCK) cells in culture. Viral lysates and the mock viral community (NIBSC reagent 11/242–001) were passed through a 0.22 µm Millipore polyethersulfone membrane filter (Millex) to remove debris and contaminants. Each filtrate (1 ml) was treated with 25 µl DNAse I (Thermo Scientific) and 50 µl RNAse A/T1 mix (Thermo Scientific) with 1× DNAse I buffer (Thermo Scientific) at 37 °C for 1 h 30 min to degrade extracapsular nucleic acids. Viral RNA was extracted using an RNeasy mini kit (Qiagen), passing a total of 900 µl nuclease-treated lysate through a column, and eluting into 100 µl elution buffer.

### DMSO treatment, reverse transcription and sequencing

Viral RNA samples were divided into 20 µl aliquots. DMSO was added to concentrations of 15, 50 and 90 (v/v) % for each sample, followed by 1 h and 30 min of incubation at 65 °C. DMSO was removed using a RNeasy MinElute cleanup kit (Qiagen), following the manufacturer’s instructions. An additional sample was treated by heat denaturation but not DMSO: the tube containing the RNA extraction was placed in boiling water for 5 min [18]. Following this, all samples were placed on ice until cDNA synthesis was carried out. Other than heat or DMSO treatment, the treatment of all samples followed standard cDNA synthesis methods.

First-strand cDNA synthesis was carried out as described in the SuperScript III first-strand cDNA synthesis kit (Fisher) instructions, by adding 5 µl each RNA sample (including a control that had not undergone DMSO treatment or column clean-up) to 1 µl random hexamer oligos, 1 µl dNTPs and 3 µl DEPC-treated water. Reactions were incubated at 65 °C for 5 min, then placed on ice for 1 min. Reverse transcriptase buffer (1×), 5 mM MgCl_2_, 0.01 M DTT, 1 µl RNAseOUT and 1 µl SuperScript III RT enzyme were added to each reaction to a total volume of 20 µl. Reactions were incubated in a thermal cycler at 25 °C for 10 min, 50 °C for 50 min and 85 °C for 5 min. Second-strand synthesis was carried out by adding 1 µl dNTPs, 0.5 µl DNA ligase, 2 µl DNA polymerase I, 0.5 µl RNAse H in 1× second-strand synthesis buffer, made up to a total volume of 40 µl with nuclease-free water. Reactions were incubated at 16 °C for 5 h and cDNA was purified with a NucleoSpin gel and PCR clean-up kit (Macherey-Nagel). Libraries were prepared for Illumina sequencing using the Nextera XT DNA sample preparation kit (Illumina), with a 1/5 ‘scaled’ library preparation protocol after that of Baym *et al*. [19].

### Bioinformatics

Reads were trimmed for adapters and quality using Cutadapt [20] and Sickle [21]. Due to a short fragment size, reads overlapped and so paired-end libraries were merged into single-end libraries using pear [22]. For Φ6 and influenza virus lysates, these libraries were mapped to the reference genomes using Bowtie2 [23], and Bam-readcount [24] was used to determine the read depth at each position. Plots were generated using the ggplot2 package in R.

For the mock viral community, a custom virus discovery pipeline was used to analyse sequencing reads [25]. Reads were translated and aligned to a viral proteome database (consisting of all annotated full or near-full viral genomes) using blastx. The significant hits to the virus database were then aligned to a non-virus-non-redundant (NVNR) universal proteome database using blastx. Hits with a more significant *E* value to NVNR than to the virus database were removed.

To test whether DMSO treatment had any effect on sequencing fidelity, we used two approaches to estimate sequencing error rates. First, we used Freebayes [26] to generate a vcf file containing all differences from the reference genome with a frequency of <5 % and a Phred quality score of at least 30. A custom Python script was used to count the number of these mutations and the total bases sequenced for each sample, and to calculate the true error rate. We tested for statistically significant differences in error rates among DMSO treatments using a proportion test. Because reference-based approaches to error estimation face limitations, we implemented a reference-free approach to error estimation [27]. We calculated the error rate for each of our DMSO treatments, as implemented in the R package ShadowRegression, and tested for differences using robust linear regressions.

## Results and Discussion

Our findings show that DMSO treatment has a dramatic effect on dsRNA sequencing (Table 1). When we prepared viral lysates for sequencing without DMSO, we did not obtain sufficient reads to cover the entire Φ6 genome. Treatment with 15  % DMSO increased the number of mapping reads over sevenfold, whilst the 50 and 90 % treatments increased the number of reads by over two orders of magnitude, allowing the full genome to be sequenced at high coverage (mean read depth of 1727 for 50 % DMSO and 1493 for 90 % DMSO).

**Table 1.** The total number of reads generated during sequencing of influenza and Φ6 after treatment at varying DMSO concentrations, and the number of those reads that mapped to reference genomes

DMSO (%)	Total no. of reads	Reads that map to Φ6	Reads that map (%)
0	1 551 220	1 377	0.1
15	1 314 520	10 358	0.8
50	1 077 133	263 993	24.5
90	1 393 569	224 562	16.1

These increases in genome coverage occurred despite very low starting nucleic acid concentrations. We used a Qubit RNA high sensitivity assay kit to quantify RNA immediately after extraction; in all cases RNA was undetectable and so assumed to be under the kit’s limit of detection of 5 ng µl^−^^1^. Additionally, we used a Qubit DNA high sensitivity assay kit to quantify the amount of cDNA synthesized. Despite this kit’s lower limit of detection of 200 pg µl^−^^1^, DNA was still not detected. Thus, for dsRNA, the raw quantity of starting material may not be as important as the efficiency of cDNA synthesis, a fact that should accounted for when preparing quality-control thresholds before next-generation sequencing.

We next sought to determine whether DMSO treatment affected other types of RNA present in the sample, which would occur in metagenomes, clinical samples or transcriptomes. DMSO treatment did not appear to affect the recovery of ssRNA-derived reads from the influenza virus genome. There was no discernible effect when the DMSO concentration was varied (Table 1). The number of reads mapping to the reference genome did decrease from the 0 % DMSO treatment to the 15 % DMSO treatment. However, this loss in mapping reads was most likely caused by the extra column clean-up step required to remove the DMSO (note that the total number of reads also decreased). We note that this decrease in reads in the influenza virus sequencing was less than one order of magnitude and still resulted in extremely high read depth, with a mean of over 1000× for every influenza segment after DMSO treatment (Table 2). This effect was obscured in Φ6 due to the large increase in mapping reads from DMSO treatment.

**Table 2.** The mean read depth for each influenza segment under varying concentrations of DMSO

DMSO (%)	Total no. of reads	Reads that map to influenza	Reads that map (%)
0	1 614 418	931 915	57.7
15	1 369 886	353 752	25.8
50	1 510 801	295 244	19.5
90	1 257 059	446 549	35.5

**Fig. 1.**
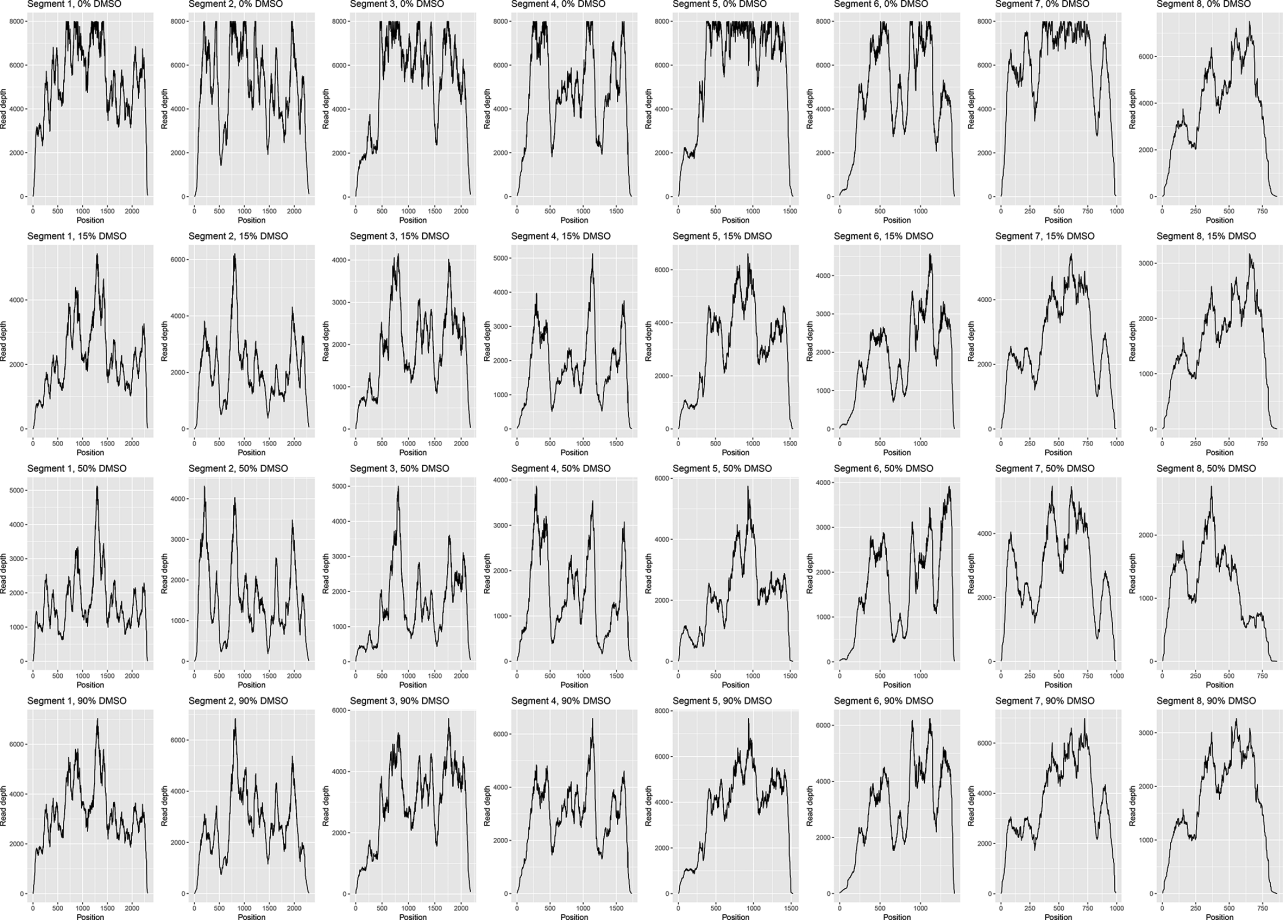
DMSO treatment does not affect sequencing read coverage across the ssRNA influenza genome. The read depth at each position in the influenza genome under varying concentrations of DMSO is shown. Note that 8000 is the maximum read depth supported by the SAM/BAM file format, so some peaks in the 0 % DMSO plots have been truncated.

**Fig. 2.**
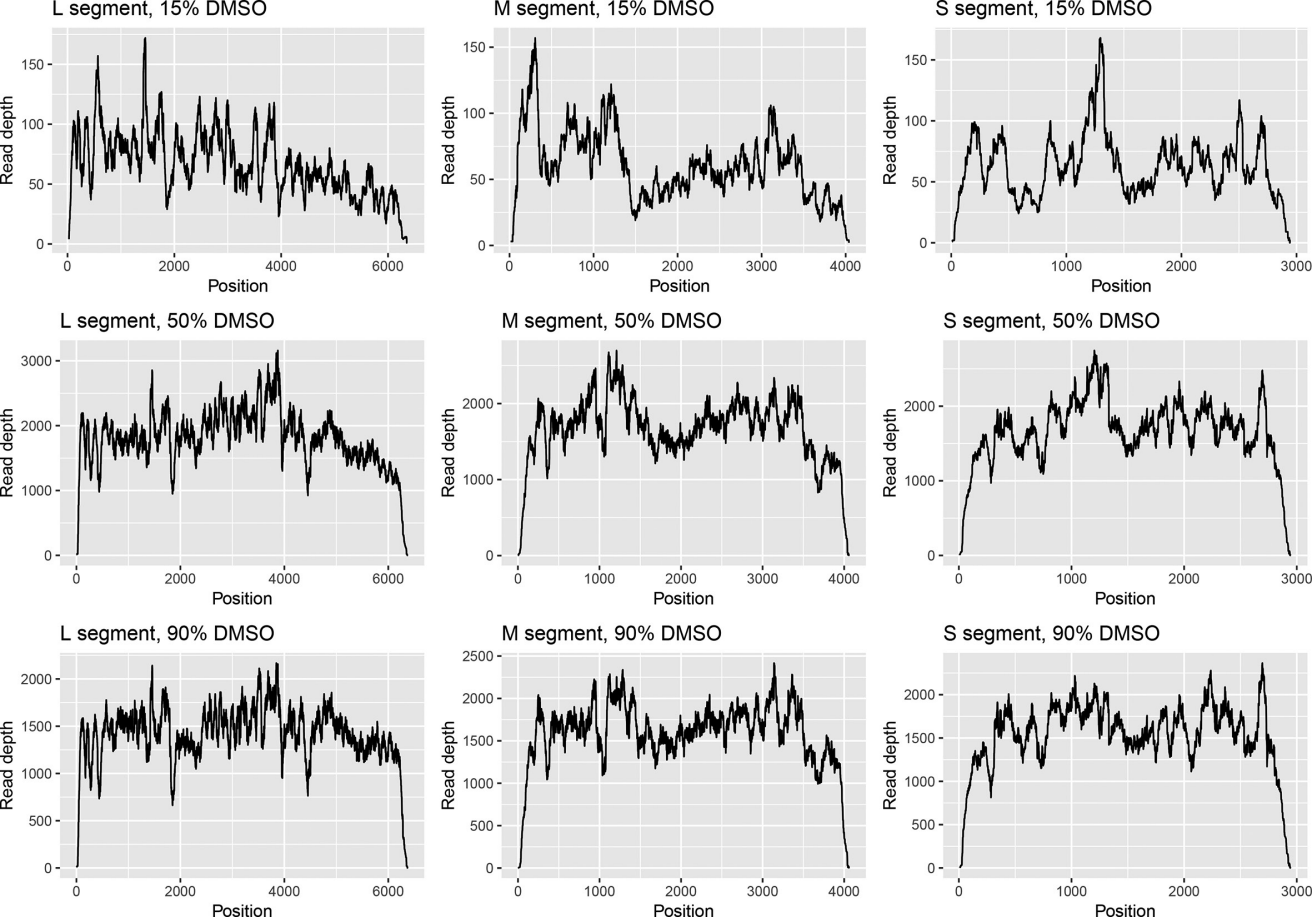
DMSO treatment greatly increases sequence coverage of the dsRNA Φ6 genome. Read depth at each position in the Φ6 genome under varying concentrations of DMSO is shown. There were insufficient reads to generate a plot for 0 % DMSO.

In order to determine whether the presence of DMSO affected any other properties of the RNA when used for downstream sequencing, we plotted the coverage at every nucleotide position for each DMSO concentration used. The plot for influenza (Fig. 1) showed a distinct, repeating pattern for each segment, with DMSO concentration appearing to have no effect on relative coverage. This indicates that there is no bias in which reads are affected by the DMSO treatment, and that the reads generated are still representative. A similar pattern could be observed for the 50 and 90 % DMSO treatments of Φ6 (Fig. 2; the low number of reads made this pattern harder to discern in the 15 % treatment). Therefore, DMSO treatment did not adversely affect genome-wide coverage patterns of dsRNA or ssRNA viruses.

**Fig. 3.**
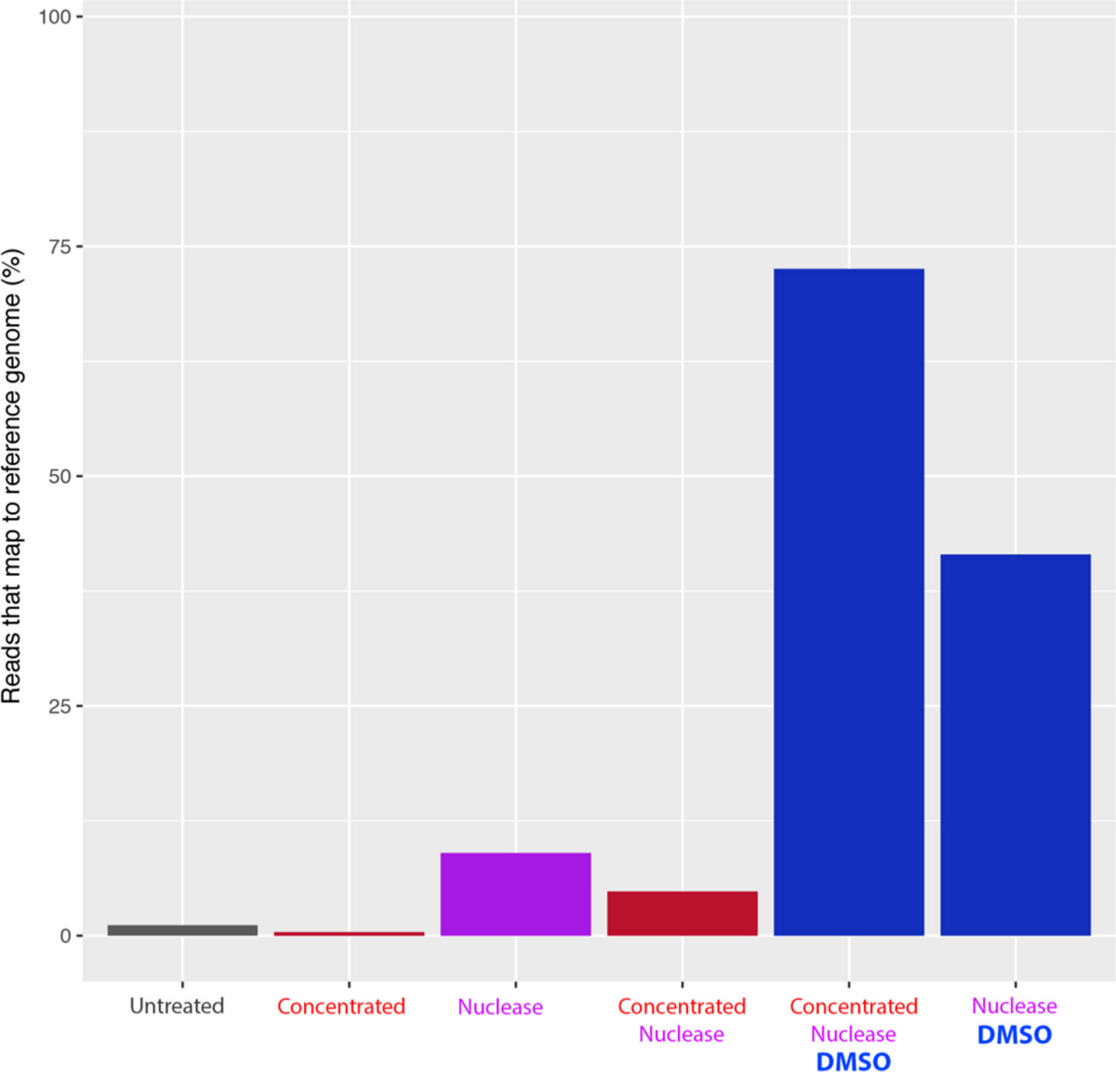
DMSO treatment has a greater effect than RNA concentration on generating dsRNA reads using next-generation sequencing. The percentages of sequencing reads mapping to the Φ6 reference genome are shown with no treatment (Untreated), concentration (Concentrated), nuclease treatment (Nuclease), nuclease treatment and concentration (Concentrated Nuclease), nuclease and DMSO treatments and concentration (Concentrated Nucelase DMSO), and nuclease and DMSO treatments (Nuclease DMSO).

To determine whether this method worked with higher starting concentrations of RNA, we used an Amicon centrifugal filter unit to concentrate approximately 10 ml Φ6 lysate into 50 µl. The concentrated lysate contained 37.6 ng RNA µl^−^^1^ (measured by Qubit) and was prepared for sequencing using 90 % DMSO (as above), as well as a control without DMSO (heat treated for 90 min at 65 °C). While only 0.08 % of reads mapped to the reference genome in the non-DMSO treated control, there was an increase to 72.45 % in the DMSO-treated sample, again demonstrating the importance of the DMSO treatment over raw RNA concentration (Fig. 3). The concentrated sample had a much higher proportion of reads than the non-concentrated sample (16.1 %; Table 1), most likely due to the concentration step increasing the ratio of viral RNA to extracapsular RNA. Therefore, DMSO treatment works at varying RNA concentrations and is likely to improve any dsRNA sequencing regardless of starting concentration.

We also tested whether DMSO treatment was more effective than simple heat denaturation. We extracted RNA from Φ6 and divided it into two aliquots. One of these was treated with 50 % DMSO as described above, while the other was placed in boiling water for 5 min. After cDNA synthesis, library preparation and sequencing, we mapped the resulting reads to the Φ6 reference genome. In the heat-treated sample, 1.72 % of reads successfully mapped to the reference, whilst in the DMSO-treated sample this increased to 40.24 %. While heat denaturation is clearly of some benefit (compared to 0.08 % of reads mapped in the no-treatment control), these data demonstrate that DMSO treatment is the superior method and might be vital when working with low starting RNA concentrations.

Because the fidelity of reverse transcriptase reactions relies on base-pairing, we examined whether DMSO treatment had any effect on fidelity by searching for errors in the sequence data using two approaches. First, we extracted all bases that were different from the reference genome with Phred quality score of at least 30. This per-base quality score is equivalent to an expected error rate of 0.1%, meaning over the entire genome sequence the expectation would be 1/1000 bases to be sequencing errors. Under the assumption that all differences from the reference were errors, these data were used to calculate the true error rate. This error rate was converted to a Phred score (Table 3). If DMSO increased the sequencing error rate, we would expect our calculated Phred score to decrease as DMSO concentration is increased. There was a statistically significant difference in error rates for each DMSO treatment of Φ6 (X^2^=533.6, df=2, *P*<2.2×10^−16^); however, the error rate did not increase with increasing DMSO concentration (the untreated, control Φ6 sample did not have enough reads for comparison). Similarly, for influenza, the difference in error rates was statistically significant (X^2^=575.08, df=3, *P*<2.2×10^−16^), but numerically miniscule (Table 3). Moreover, the error rate decreased as the DMSO concentration increased. In all cases, the true error rate was lower than the expected error rate. Additionally, our calculated error rate is conservative, because of the assumption that all differences from the reference were erroneous. In actuality, some of these differences at low frequency could be true mutations in RNA virus populations (i.e. minor variants).

**Table 3.** The error rate and quality score for each of the Φ6 and influenza samples treated with DMSO. The error rate was not determined for Φ6 0% DMSO, because this treatment generated insufficient reads (see Table 1).

Organism	DMSO (%)	Total no. of errors	Total no. of bases sequenced	Error rate (%)	Phred quality score
Φ6	0	not determined	not determined	not determined	not determined
15	176	857 974	0.021	36.9
50	8 940	23 127 006	0.039	34.1
90	5 276	19 996 871	0.026	35.8
Influenza	0	34 527	68 561 988	0.050	33.0
15	13 181	29 087 984	0.045	33.4
50	9 549	23 120 765	0.041	33.8
90	16 757	40 369 161	0.042	33.8

**Fig. 4.**
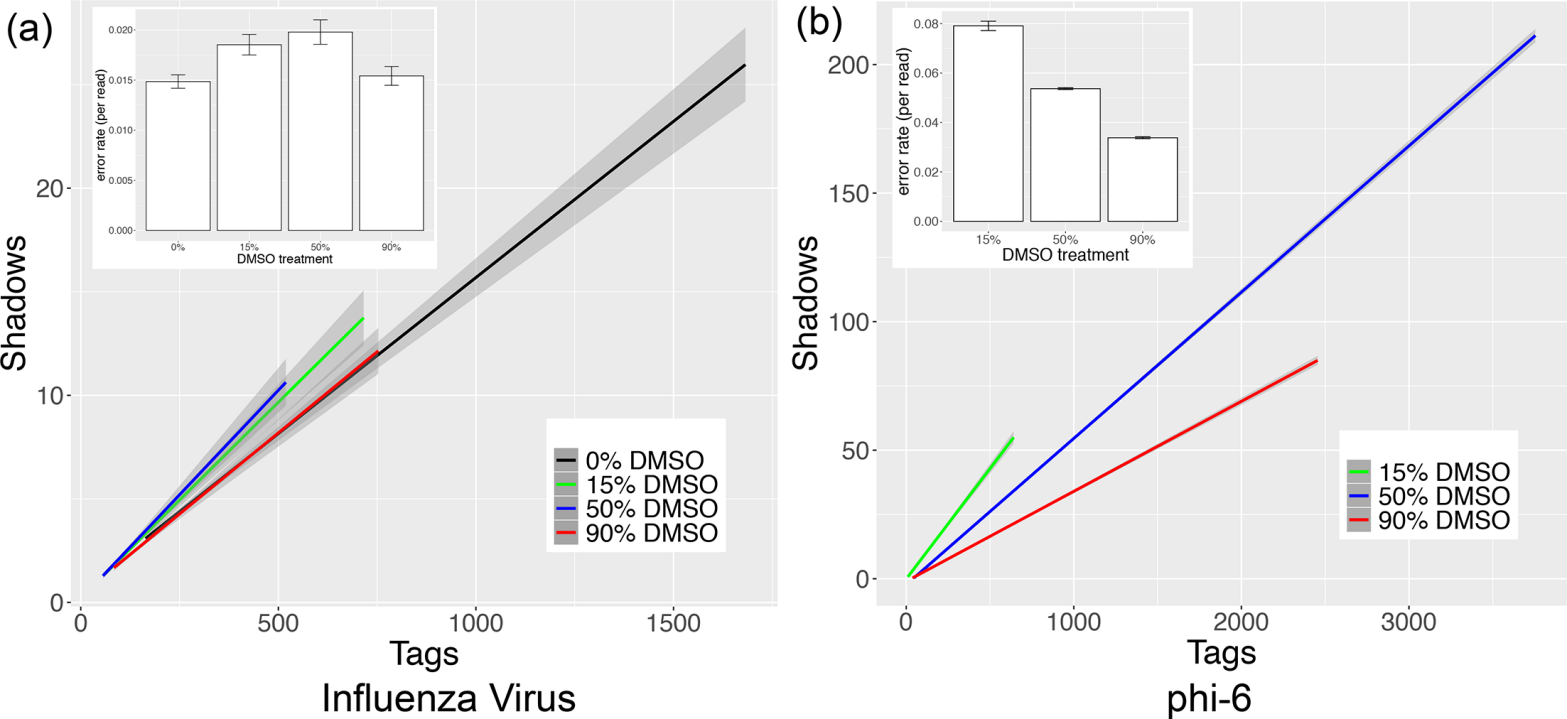
DMSO treatment does not adversely affect sequencing error rates in influenza (a) or Φ6 (b). The R package ShadowRegression estimates reference-free error rates (inset) based on a transform of the slope of read counts and their ‘shadows’ (main plot line graphs).

Second, to estimate error rates in short reads without a reference genome, we also used the ShadowRegression R package [27], which compares reads against each other. Our data show that DMSO has little effect on the error rate of the ssRNA influenza virus (Fig. 4). Using a robust F-test, we found that the difference in influenza error rate between each DMSO-treated sample and the untreated sample was not statistically significant (15% DMSO, F=1.4003, *P*=0.134; 50 % DMSO, F=2.246, *P*=0.2367; 90 % DMSO, F=0.2093, *P*=0.6473). Moreover, these error rates did not show any pattern with increasing DMSO concentration (Fig. 4a, inset). However, in Φ6 we found that as the concentration of DMSO increased, the sequencing error rate actually decreased (Fig. 4b, inset). This decrease compared to the 15 % DMSO treatment was statistically significant (50 % DMSO, F=280.95, *P*<2.2×10^−16^; 90 % DMSO, F=37.391, *P*=1.092×10^−9^). Thus, using two statistical approaches, these data collectively indicate that DMSO did not have an adverse effect on read accuracy and in some instances may improve sequencing accuracy.

The mock viral community (NIBSC reagent 11/242–001) was made by mixing 25 eukaryotic viruses [17]. Of those with RNA genomes, the most abundant in the sample is thought to be the dsRNA rotavirus, based on real-time PCR results [25], which we selected for comparison with the ssRNA virus human parechovirus 3. Using a virus discovery pipeline, we only detected 1 read from a dsRNA virus (rotavirus A) in the sample untreated with DMSO (Table 4). However, under the 15 % DMSO treatment, this increased to over 203 rotavirus reads, as well as reads from dsRNA viruses not thought to have been in the reagent, including human picobirnavirus and some totiviruses. This result is notable because the mock viral community was made such that rotavirus A was the most abundant virus [25]. If the most abundant virus can be so under-represented in a known sample, this suggests many metagenomic studies will miss almost all dsRNA viruses. Furthermore, we indeed find dsRNA viruses that had previously evaded detection in the mock viral community [17]. The 50 and 90 % DMSO treatments contained significantly fewer dsRNA reads than the 15 % treatment, but reads from all viruses were reduced in these samples. Although the number of reads detected in the DMSO-treated samples were low compared with the untreated sample, dsRNA was practically undetectable in the latter. Therefore, despite some caveats, we suggest that the power of this method for detecting dsRNA viruses in metagenomic samples is clear.

**Table 4.** Total number of reads detected in reagent 11/242–001 under each DMSO treatment for human parechovirus, rotavirus A and other dsRNA viruses

DMSO (%)	Parechovirus hits	Rotavirus A hits	Other dsRNA virus hits
0	3263	1	0
15	2089	203	27
50	293	10	9
90	785	11	4

### Conclusion

Sequencing of dsRNA can frequently be problematic, with traditional cDNA synthesis being highly inefficient. We have demonstrated that a simple treatment with a cheap and common laboratory reagent can increase the number of sequencing reads from dsRNA organisms by over two orders of magnitude. Importantly, the positive effect of the DMSO treatment occurred independent of RNA concentration, even when RNA was undetectable. Furthermore, DMSO treatment was more important than RNA concentration in determining dsRNA read yield and it did not affect viral genome coverage. We suggest that samples to be sequenced that contain or are suspected to contain dsRNA are treated with at least 50 % DMSO prior to cDNA synthesis. This treatment should also improve sequencing of dsRNA involved in innate immunity in plants and animals. We suspect this treatment can be successfully applied to other DNA sequencing technologies, because the DMSO treatment occurs at the cDNA synthesis step and has been shown to improve other procedures, such as Sanger sequencing [12].

When preparing an environmental sample for sequencing, it is possible that there may be dsRNA viruses present that are undetectable when following standard protocols. Previous data have shown dsRNA viruses to be under-represented in metagenomic samples [25]. Our data on the mock viral community (where the putatively most abundant virus was not detected without DMSO treatment) suggests dsRNA viruses will almost invariably go undetected in environmental samples. We have shown that not only will DMSO treatment increase representation of these organisms, but also the effect on ssRNA representation is minor. It may well be that dsRNA viruses are more numerous than thought, but remain undetected using traditional methods.

## Data bibliography

1.Sequence data: Wilcox, AH, Delwart E, Díaz-Muñoz SL. National Center for Biotechnology Information Short Read Archive (accession numbers: PRJNA527100, PRJNA527101, PRJNA527098) (2019).

2. Wilcox, AH, Delwart E, Díaz-Muñoz SL. Code and data: GitHub – https://github.com/awilcox83/dsRNA-sequencing/ (doi:10.5281/zenodo.3530803) (2019).
